# Partial versus total knee arthroplasty for isolated antero-medial osteoarthritis – An analysis of PROMs and satisfaction

**DOI:** 10.1051/sicotj/2023006

**Published:** 2023-04-21

**Authors:** Adarsh Annapareddy, Praharsha Mulpur, Mrinal Prakash, A. B. Suhas Masilamani, Krishna Kiran Eachempati, A. V. Gurava Reddy

**Affiliations:** 1 Consultant Orthopaedic and Joint Replacement Surgeon, Sunshine Bone and Joint Institute, KIMS-Sunshine Hospitals 500003 Hyderabad India; 2 Research Fellow, Joint Replacement Surgery, Sunshine Bone and Joint Institute, KIMS-Sunshine Hospitals 500003 Hyderabad India; 3 Consultant Orthopaedic and Joint Replacement Surgeon, Department of Orthopaedics, Medicover Hospitals 524002 Hyderabad India

**Keywords:** Unicompartmental, Arthroplasty, AMOA, Satisfaction, PROM

## Abstract

*Aim*: This study aimed to compare the patient-reported functional outcomes and patient satisfaction after medial Unicompartmental Knee Arthroplasty (UKA) versus Total Knee Arthroplasty (TKA), performed for anteromedial osteoarthritis (AMOA) of the knee in patients from an Indian population, at a minimum 3-year follow-up. *Methods*: This is a prospective matched cohort study (1:2 ratio). One hundred and one UKA cases were matched to 206 TKA cases by propensity score matching for age, body mass index (BMI), gender distribution, and the Charlson Comorbidity Index (CCI). The primary outcome (Oxford knee score, OKS) was assessed at a 3-year follow-up, along with secondary outcomes (Western Ontario and McMaster Universities Osteoarthritis Index [WOMAC] Score, Forgotten Joint Score (FJS), Anterior Knee Pain (Kujala) score, patient satisfaction, and revision rate at the final follow-up). *Results*: The UKA group was superior to the TKA group in patient-reported functional outcomes based on the OKS (*p* = 0.004). Using the FJS score, UKA was more likely to be a forgotten joint compared to TKA (*p* < 0.001). However, differences in the OKS and FJS did not meet the reported minimal clinically important difference (MCID) thresholds. Quality of life (EuroQol-5D VAS scale) was found to be significantly higher in the UKA group (*p* < 0.001). Patients in the UKA group were more likely to be very satisfied (75.2%) versus the TKA group (62.1%, *p* = 0.023). *Conclusion*: For AMOA, UKA was associated with improved patient satisfaction compared to TKA. Although patient-reported outcome measures were statistically in favour of UKA over TKA, the differences were not clinically significant. Multicenter and randomized studies comparing the two procedures are warranted.

**Evidence**: Level-II Therapeutic

## Introduction

Total knee arthroplasty is one of the most successful surgeries in the management of advanced osteoarthritis (OA) of the knee. White et al. [1] reported anteromedial osteoarthritis (AMOA) as a distinct pattern of wear, characterized by isolated and complete loss of medial knee cartilage, without wear in the lateral or patellofemoral compartment.

The prevalence of isolated medial compartment OA is reported to be as high as 51.2% in the Caucasian population [2]. A recent study has shown a high prevalence (46.94%) of isolated AMOA even in Indian patients undergoing primary TKA for OA of the knee [3]. However, the utilization of UKA remains low, with registry data indicating TKA rates of over 80% [4, 5].

While TKA is associated with lower revision rates compared to UKA [6, 7], UKA offers the advantages of faster rehabilitation, reduced blood loss, and fewer peri-operative complications compared to TKA [8–12]. Survivorship is often an important factor in decision-making for TKA versus UKA [13].

Previous studies have evaluated differences in objective functional outcome scores (Knee Society Score or HSS score) and most studies have reported comparable outcomes [11, 13–15]. Only a few studies have reported patient-reported outcomes (PROMs) [16–18]. To the best of our knowledge, there is only one study published, that objectively compares patellofemoral symptoms, reporting the incidence of anterior knee pain and the Anterior Knee Pain Score (AKPS), after either procedure [18].

There are no studies that have compared PROMs and satisfaction, between UKA and TKA for isolated AMOA in the Indian population. The primary aim of this study was to compare PROMs based on the Oxford Knee Score (OKS) between UKA and TKA in an Indian patient population with isolated medial compartment arthritis of the knee. The secondary objectives include a comparison of the forgotten joint score (FJS), quality of life index (EQ-5D VAS), patellofemoral symptoms (Kujala score) and patient-reported satisfaction among the two groups

## Material and methods

This study is a prospective propensity-matched cohort study of patients with AMOA of the knee, who underwent either a medial UKA or a TKA at a single high-volume arthroplasty centre between 2017 and 2018. The study was approved by the Institutional Ethical Committee and was conducted according to the guidelines of the Declaration of Helsinki [19]. Patients with a minimum follow-up duration of 3 years were included.

During the study period, a total of 2518 patients underwent knee arthroplasty at our institute. Of these patients, 760 patients (30%) were diagnosed clinically and radiologically with AMOA of the knee. These 760 patients were counselled to undergo UKA or TKA; accordingly, 120 patients consented to UKA and 640 patients consented to TKA for AMOA. After intra-operative exclusions based on signs suggestive of inflammatory arthropathy, patellofemoral arthritic wear, and ACL status, 101 patients underwent UKA, and 580 patients underwent TKA.

### Matching criteria

**Figure 1:**
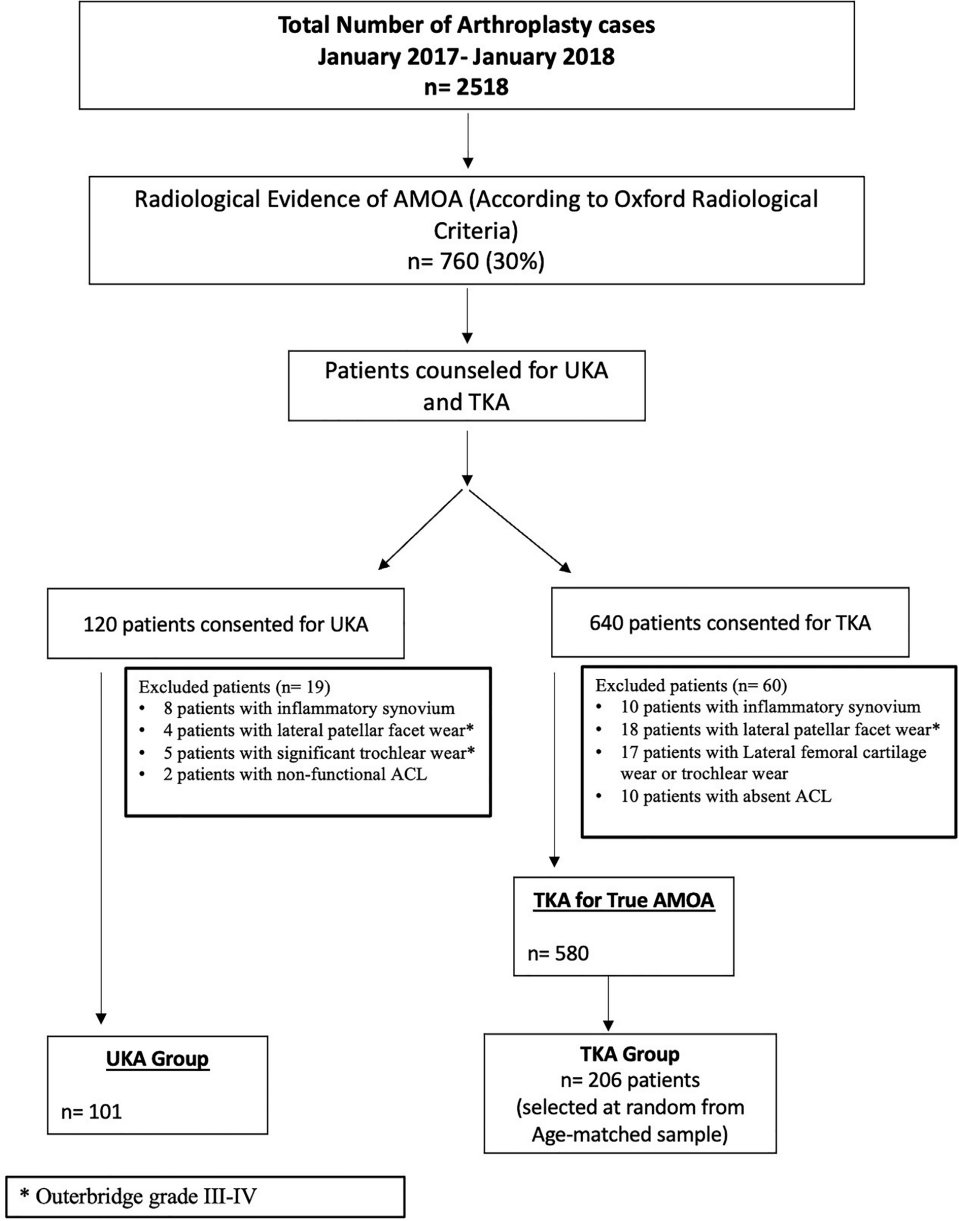
Flowchart for case allocation in the UKA and TKA cohorts. *Patients excluded based on the Outerbridge Classification of cartilage wear of the patello-femoral Joint.

Patients in the TKA group were selected by propensity-score-matching. The variables used to derive the matched group included age, gender, body mass index (BMI), pre-operative functional status based on the OKS score, and the Charlson Comorbidity Index (CCI) by logistic regression. The case recruitment flowchart is illustrated in Figure 1. After matching the UKA and TKA groups, the final analysis included 101 patients in the UKA group and 206 matched patients in the TKA group.

### Diagnostic criteria and surgical indications

All adult patients who presented to the outpatient department with symptoms suggestive of primary OA of the knee were evaluated by a senior consultant arthroplasty surgeon; they subsequently underwent radiological evaluation, using the following radiographs:

1. Standing radiographs of the knee (anteroposterior [AP] and true lateral views).
2. Stress radiographs of the knee at 20 degrees of knee flexion (Varus and Valgus stress views).
3. Skyline view of the patellofemoral joint.

The radiographs obtained were screened by the consultant and a research fellow and evaluated based on the Oxford Partial Knee Arthroplasty radiological decision guide to diagnosing AMOA [20]. Patients diagnosed with AMOA were counselled for both UKA and TKA.

Patellofemoral joint (PFJ) wear was also evaluated by the Oxford radiological decision aid. The PFJ wear was considered “acceptable” in cases with a normal joint space or isolated medial facet OA (with or without bone loss) or lateral facet OA without bone loss. PFJ wear was considered “unacceptable” if it was associated with lateral facet OA associated with bone loss, grooving, and subluxation. Those patients with clear radiological evidence of advanced PFJ arthritis were not eligible for inclusion in the study. Only patients with BMI less than 40 kg/m^2^ were included in this study.

For patients with radiological evidence of AMOA, diagnosis of AMOA and eligibility for UKA were again confirmed intra-operatively using the following criteria, which were documented photographically:

1. Intact ACL.
2. Functional ACL with linear striations or fibrillations.
3. Normal lateral compartment cartilage.
4. Anteromedial OA on the resected tibial biscuit.
5. Absence of lateral patellar facet arthritis/cartilage loss over the trochlea.

**Figure 2:**
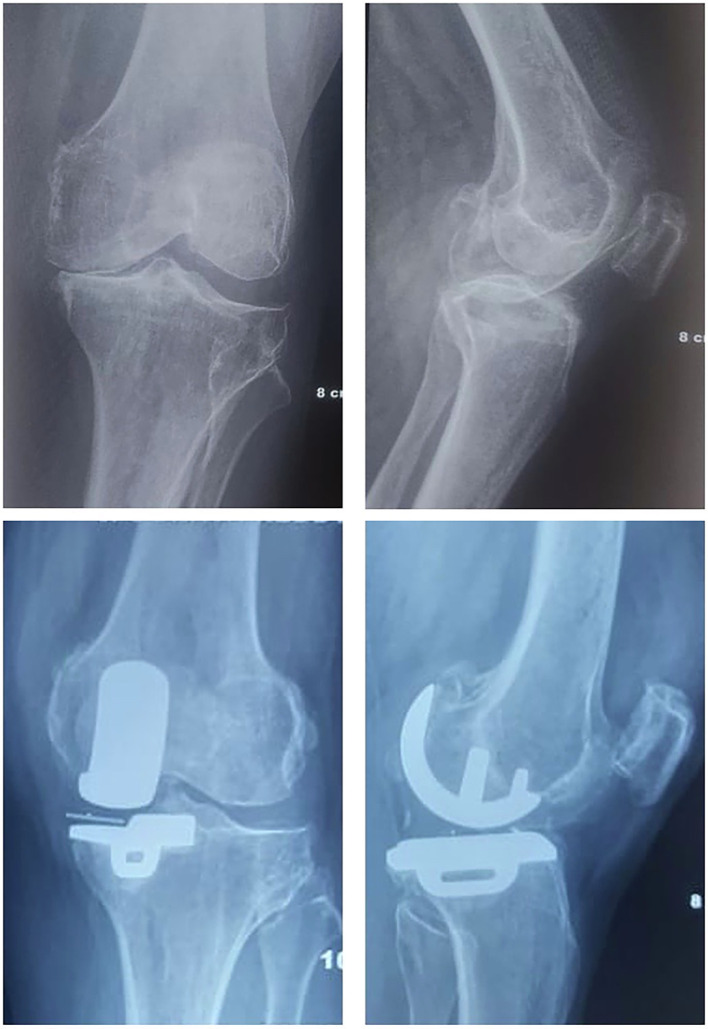
Pre- and Post-operative radiographs of Oxford mobile bearing UKA for medial OA of the knee.

**Figure 3:**
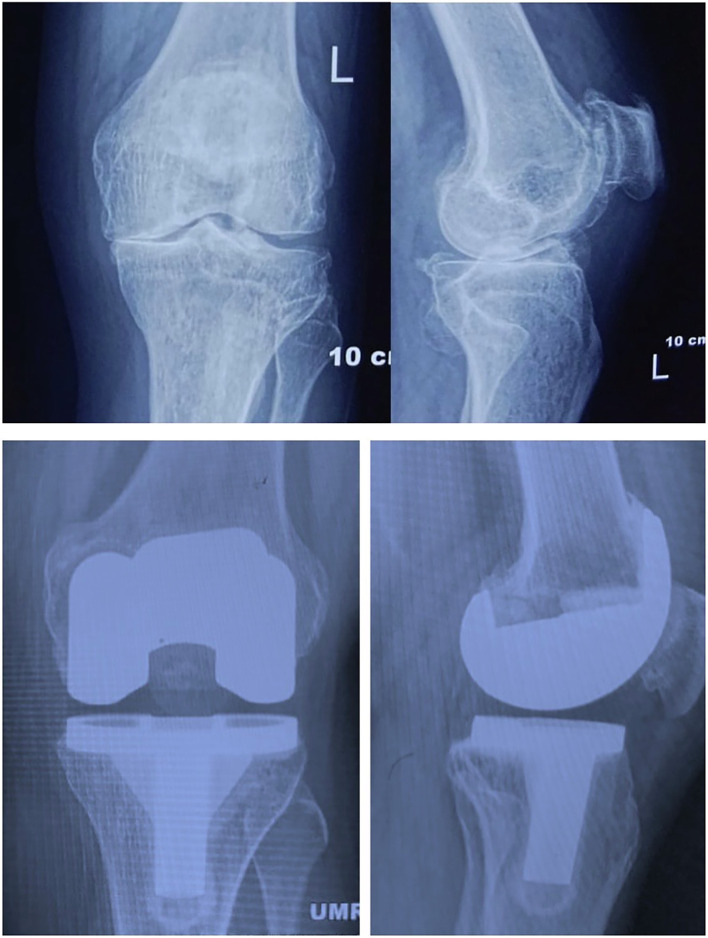
Pre- and Post-operative radiographs of TKA (PFC Sigma-PS) for medial OA of the knee.

In all cases (UKA and TKA), the patellar cartilage wear was evaluated intra-operatively and classified according to the Outerbridge classification. Patients undergoing UKA or TKA, with Outerbridge arthritic grade 0–II, were considered for inclusion in the study while those with advanced patellofemoral joint wear (Outerbridge grades III–IV) were excluded from both groups. Patients undergoing UKA received a cemented medial unicompartmental mobile-bearing Oxford prosthesis. Patients undergoing TKA received a cemented posterior-stabilized DePuy PFC-Sigma prosthesis, without resurfacing the patella in all cases (Figures 2 and 3). All surgeries were performed by a single senior arthroplasty surgeon (A.V.G.R), who has been trained and certified in Oxford UKA. Patients were followed-up at 3, 6, and 12 months after surgery, and annually, thereafter.

### Follow-up outcomes assessed

The primary outcome measure was the patient-reported Oxford Knee Score (OKS). Secondary outcome measures included the Western Ontario and McMaster Universities Osteoarthritis Index (WOMAC) Score, Forgotten Joint Score (FJS), Anterior Knee Pain (Kujala) score, patient satisfaction and revision rate at final follow-up. Satisfaction at the final follow-up was assessed with the question “How satisfied are you with your surgery” and responses were graded on a 4-point Likert scale (1 = Very satisfied; 2 = Somewhat Satisfied; 3 = Dissatisfied; and 4 = Very Dissatisfied). A revision was defined as re-operation for any reason after index surgery. The mean differences in PROMs (OKS, FJS, WOMAC, EQ-5D VAS) were compared against the published minimal clinically important difference (MCID) thresholds to evaluate the clinical significance of our outcomes [21–24].

## Statistical analysis

Sample size estimation was performed *a priori*. Assuming 80% power and 5% (0.05) significance to detect a 5-point difference (MCID) in the Oxford Knee Score, it was estimated that a total of 140 patients (70 in each group) were required to adequately power the analysis. Baseline characteristics were compared between both the groups; the Chi-square test was applied for categorical variables and the independent samples *t*-test for continuous variables. Normally distributed data were evaluated using independent samples *t*-test and non-parametric data were evaluated using the Mann–Whitney *U*-test. All the results are presented as mean (with standard deviation [SD]) and 95% confidence intervals (CI). A *p*-value less than 0.05 was considered statistically significant.

## Results

A total of 307 subjects were included in the final analysis, with UKA:TKA in a 1:2 matching ratio (UKA or Study group 101 subjects and TKA or control group with 206 patients). These groups were age-matched before analysis. The demographic and baseline characteristics of both groups are summarized in Table 1. The minimum follow-up duration was 3 years in both groups.

**Table 1:** Baseline characteristics of the study participants.

Scores	UKA group		TKA group		*p*-value
	Mean	SD	Mean	SD	
Age	61.32	9.1	60.65	7.9	0.505*
Charlson Comorbidity Index (CCI)	2.42	1.4	2.11	1.2	0.06*
Body mass index (kg/m^2^)	28.49	4.1	28.45	4.8	0.933*
Pre-operative varus deformity (in degrees)	6.84	2.9	7.06	3.8	0.254*
Pre-operative knee flexion (in degrees)	107.52	8.1	103.01	7.8	0.672*
Mean follow-up (in years)	3.4	1.9	3.8	2.5	0.667*
Pre-operative Oxford Knee Score	21.7	5.8	20.6	5.3	0.147*

* Mann–Whitney *U*-test.

## Analysis of outcomes

The comparison of outcomes is summarized in Table 2. Analysis of patient satisfaction after surgery is summarized in Table 3.

**Table 2:** Summary of outcomes at final follow-up.

Variable	UKA group (*n* = 101)	TKA group (*n* = 206)	*p*-value*
Median OKS (IQR)	42 (40–44)	40 (38–42)	**<0.001**
Mean OKS (SD)	41.14 (5.8)	39.32 (3.2)	**0.004**
Median FJS (IQR)	67 (67–84)	62 (55–76)	**<0.001**
Mean FJS (SD)	72.1 (15.7)	61.5 (8)	**<0.001**
Median WOMAC (IQR)	6 (5–7)	8 (6–10)	**<0.001**
Mean WOMAC (SD)	7.89 (6.51)	8.47 (2.6)	0.390
Median EQ 5D VAS (IQR)	80 (80–85)	74 (70–80)	**<0.001**
Mean EQ 5D VAS (SD)	81.5 (6.6)	74.3 (7.1)	**<0.001**
Median Kujala (IQR)	70 (68–75)	70 (68–71)	0.303
Mean Kujala Score (SD)	69.3 (6.47)	68.8 (3.4)	0.484

* Mann–Whitney *U*-test.

SD = Standard deviation, UKA – Unicompartmental knee arthroplasty, TKA – Total knee arthroplasty, IQR – Inter-Quartile Range.

Values in bold are statistically significant.

**Table 3:** Comparison of patient satisfaction.

Patient satisfaction	UKA group (*n*, %)	TKA group (*n*, %)	*p*-value
	(*n* = 101)	(*n* = 206)	
Very satisfied	76 (75.2)	128 (62.1)	0.023*
Satisfied	20 (19.8)	69 (33.5)	0.01*
Dissatisfied	5 (5)	9 (4.4)	0.6883*

* Proportional chi-squared test.

### Patient-reported outcomes (PROMs)

**Figure 4:**
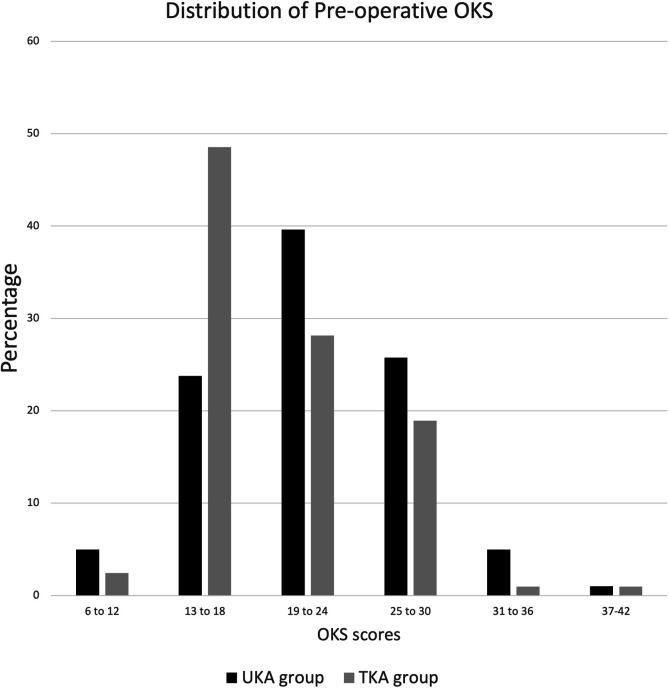
Histogram depicting the distribution of the pre-operative Oxford Knee Score of both the cohorts.

**Figure 5:**
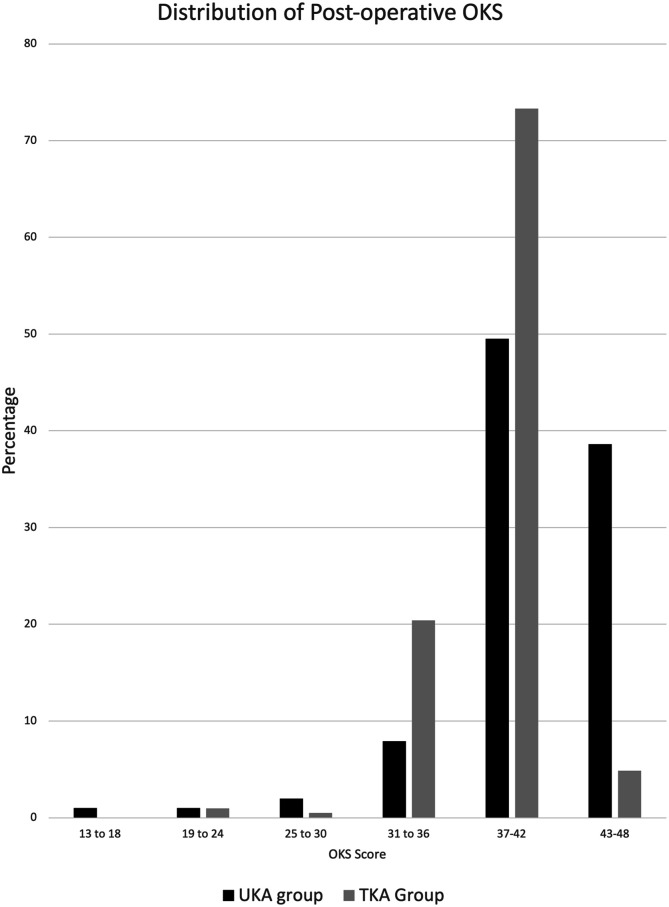
Histogram depicting the distribution of the post-operative Oxford Knee Score of both the cohorts.

Both the groups showed a significant improvement from the pre-operative OKS [(UKA group: Mean [±SD] pre-op OKS 21.7 [±5.82]); (TKA group: Mean pre-op OKS 20.6 [±5.36])] to the post-surgery OKS at latest follow-up [(UKA group: Mean post-op OKS 41.14 [±5.81], Mean difference of 19.44 points, *p* < 0.001); (TKA group: Mean post-op OKS 39.32 [±3.20], Mean difference of 19.67 points, *p* < 0.001). There was no significant difference between the mean improvement observed with either surgery. The percentage distribution of the pre-operative and post-operative OKS is represented in Figures 4 and 5 respectively.

The Patient Acceptable Symptomatic State (PASS) cut-off for the Oxford Knee Score was set at 37-points (out of the maximum 48) and the MCID for the OKS was set at 5-points as the cut-off, based on published reports [25–27]. A higher proportion of patients met the PASS criteria or cut-off in the UKA group; 88.8% (*n* = 88/100) compared to 78% (*n* = 161/206) in the TKA cohort. With an MCID cut-off of 5-points, only 2 (2%) of the UKA cohort and 5 (2.4%) did not meet the cut-off.

Medial UKA was more likely to be a forgotten joint, with a significantly higher FJS-12 score (Mean [±SD] FJS 72.1 [±15.7]) compared to TKA (Mean [±SD] FJS 61.5 [±8], *p* < 0.001). However, the mean difference (10.6) falls short of the published MCID for the FJS (13.7); the clinical relevance of the findings should be interpreted accordingly [22].

Functional outcomes based on the WOMAC score were comparable between both groups. The mean cumulative WOMAC score in the UKA group was lower, indicating better functional outcome (Mean [±SD] WOMAC score in the UKA group: 7.89 [±6.51]; Mean [±SD] WOMAC score in the TKA group: 8.47 [±2.6], *p* = 0.390). The mean difference was lower than the MCID for the WOMAC score and is not clinically significant.

There was no difference in patellofemoral outcomes between the two cohorts. The mean Kujala scores were comparable (UKA: Mean [±SD] Kujala score: 69.3 [±6.47] vs. TKA: Mean [± SD] Kujala score: 68.8 [±3.4], *p* = 0.484). The difference in the EQ-5D VAS scale for quality of health was statistically and clinically significant, with a mean EQ-5D VAS of 81.5 (±6.6) in the UKA cohort compared to 74.3 (±7.1) in the TKA cohort, with a mean difference of 7.2.

Patient-reported satisfaction was higher in patients undergoing medial UKA. A significantly higher proportion of patients were very satisfied or satisfied after UKA (76% in UKA versus 62.1% in the TKA group, *p* = 0.023, Table 3).

There were no revisions or re-operations for any cause in either group at the final minimum follow-up of 3 years.

## Discussion

With an ever-increasing volume of primary knee arthroplasty, surgeons are often faced with patients with early or isolated medial arthritis of the knee [28]. The debate of UKA versus TKA for the management of AMOA has been long-standing. Most published studies are retrospective or underpowered and are majorly reported in the Caucasian population [14, 18, 29–38]. Only a few studies have incorporated PROMs in the outcomes assessed [29, 31–33, 39, 40]. No studies are comparing patient-reported outcomes along with patellofemoral outcomes and satisfaction after UKA or TKA for AMOA in the Asian ethnicity. The most cited publications comparing UKA versus TKA for medial OA are summarized in Table 4.

**Table 4:** Literature published comparing outcomes after UKA versus TKA for isolated medial arthritis of the knee.

Author	Study design	Outcome measures	Study population	Remarks
Casper et al. [14]	Multicentre, Retrospective, 2-year follow-up	KSS-2015	83 UKA	No PROMS assessed
	Propensity score weighted cohorts		50 TKA	Only demonstrated trends in differences in sub-scores of KSS
				Improved function but equivalent KSS-Satisfaction
Sershon et al. [39]	Single centre RCT	KOOS Jr	57 UKA	Comparable early outcomes at 6-months
		KSS		
		FJS	50 TKA	Caucasian Population
		VR-12, ROM		
Kulshrestha et al. [32]	RCT	KOS-ADLS	36 UKA	Comparable in all outcome measures
		OKS	36 TKA	
		HAAS		
		Delaware index		
Amin et al. [38]	Matched Cohort Study (Retrospective)	KSS	54 UKA	No functional difference
		5-year survival	54 TKA	Better survivorship with TKA
Newman et al. [30]	RCT	BKS, Complications and Revisions	50 UKA	Comparable functional outcomes, ROM
			52 TKA	No PROMs assessed
Weale et al. [34]	RCT	VAS, ROM	50 UKA	No PROMs assessed
			52 TKA	
Costa et al. [37]	RCT, *UKA and TKA in* the s*ame patient undergoing bilateral arthroplasty*	VAS, ROM	34 UKA	Comparable functional outcomes and ROM
		Complications and revision	34 TKA	No PROMS assessed
Sun and Jia [36]	RCT	KSS	28 UKA	Comparable functional outcomes and ROM
		ROM, Complications and revision	28 TKA	No PROMS assessed
Dalury et al. [35]	Retrospective, *UKA and TKA in* the s*ame patient undergoing bilateral arthroplasty*	KSS	23 Patients	Comparable KSS at final follow up
		ROM		No PROMS assessed
Knifsund et al. [29]	RCT	OKS	69 UKA	Comparable outcomes
		KOOS	70 TKA	
Beard et al. [31]	RCT (TOPKAT Trial)	OKS	233 UKA	Similar clinical outcomes Similar complication and revision rates UKA more cost-effective
		KSS	231A	
		UCLA Score		
		EQ-5D		

This prospective study demonstrated a significantly higher patient satisfaction after UKA for isolated AMOA, compared to TKA. Both procedures were associated with a significant improvement in the OKS compared to the pre-surgery baseline. However, the differences in all PROMs assessed (OKS, FJS, WOMAC, Kujala) were comparable between both groups. Although the results of this study show statistically significant improvement in patient-reported outcomes and satisfaction in favour of UKA for AMOA, none of the reported outcome parameters reached the MCID values [21–23].

This study has some limitations. Firstly, this was a prospective matched-cohort study and not a randomized control trial. There could be an inherent bias in the selection of patients and counselling for UKA versus TKA due to the non-randomized nature of patient selection. A multi-centre RCT and meta-analysis of studies on the topic may be required to establish the superiority of one procedure over the other. Secondly, there is potential bias due to patients’ self-selection into the UKA or TKA cohorts. Another major limitation of the study is the minimum follow-up of 3 years, which is relatively short and inadequate to provide strong evidence about revision rates.

This study has several strengths. The study was adequately powered based on the primary outcome measure of PROM (OKS). All surgeries were performed by a single surgeon, and UKA and TKA surgeries were performed at a high-volume arthroplasty centre, with high adoption of unicompartmental knee arthroplasty. Both cohorts were matched by propensity score matching for age, BMI, and gender distribution. The pre-operative OKS, coronal deformity, and risk profile of patients in both cohorts were comparable, without significant differences. There was no loss to follow up in either group.

Casper et al. reported a greater improvement in the KSS-Functional sub-score in the UKA group [14]. On the contrary, TKA was associated with a better outcome on the KSS-Symptoms sub-score. There was no difference in patient-reported satisfaction. Pongcharoen et al. compared functional outcomes using performance-based tests [41]. UKA was associated with faster recovery than TKA, at 6 months. The benefit was short-lived with no difference at 1 and 2 years after surgery.

Witjes et al. reported comparable early clinical outcomes between the UKA and TKA groups, with insignificant differences in the Knee Injury and Osteoarthritis Outcomes Score (KOOS) and anterior knee pain score [18]. This was the only study we are aware of, that reported differences in anterior knee pain after UKA and TKA with non-resurfacing of the patella. They reported comparable patellofemoral scores in both groups, similar to the findings of our study.

Liddle et al. reported the findings of the national registry-based (England and Wales) study on the outcomes after UKA versus TKA at a very early follow-up of 6 months [10]. The TKA group had a higher incidence of complications, and all PROMs assessed were in favour of UKA over TKA in a propensity score-matched study population. However, the benefits do not justify the risk of higher rates of revision and the short follow-up duration of this study was a major limitation.

Mikkelsen et al. compared outcomes (OKS) and the likelihood of patients attaining an acceptable clinical state (PASS) with both UKA and TKA for AMOA [42]. They reported higher odds of patients attaining the PASS cut-off of the OKS with UKA compared to TKA. Our findings were similar, with a higher proportion of patients attaining the PASS cut-off in the UKA group compared to TKA (88.8% vs. 78%).

Patients in the UKA group were more likely to have “forgotten” the artificial joint after surgery. The mean difference in FJS-12 for both groups was 10.6 points, which was statistically significant but did not meet the reported MCID of the FJS-12 [22]. Zuiderbaan et al. reported similar findings in their prospective study of 65 patients in each group (UKA vs. TKA), with significantly higher FJS scores in the UKA group [33]. The FJS was significantly higher in the UKA group (FJS at 1 year: 73.9 ± 22.8; FJS at 2 years: 74.3 ± 24.8) in contrast to the TKA group (FJS at 1 year: 59.3 ± 29.5 (*p* = 0.002); FJS at 2 years 59.8 ± 31.5 (*p* = 0.004).

The EuroQol 5-dimensional assessment tool was used to assess the quality-of-life or health-status difference after surgery, between the two groups. The EQ-5D VAS scale is scored on a scale of 0–100. In this study, patients in the UKA group reported a significantly higher and better QoL outcome compared to the TKA group. The MCID of the EQ-5D VAS was reported as a difference of 6.9 points [24]. The UKA cohort had a higher mean EQ-5D VAS and the mean difference of 7.2 was clinically relevant and statistically significant.

A significantly higher proportion of patients were very satisfied in the UKA group compared to the TKA group (76% vs. 62.1%). Dissatisfied patients in the TKA group complained of persistent and diffuse knee pain after surgery; however, none of these patients underwent revision surgery. There were no revisions in either group. Dissatisfied patients in the UKA cohort complained of persistent knee pain. Two patients (2 out of 101) complained of knee effusions and pain; however, clinically they did not present with instability or radiological evidence of component failure and did not require re-operation.

In a National Joint Registry (NJR) study, Baker et al. reported comparable outcomes in PROMs (OKS) and quality of life [40]. This registry-based study advocated a cautious approach to the adoption of UKA, in view of the increased revision rates. On the contrary, Goodfellow et al. studied the revision rates in another NJR and reported a significant measurement bias in the reporting of revisions after knee arthroplasty [43]. They found the threshold to revise UKA with lower functional outcome scores was far lower than to revise a TKA with similar outcomes. They concluded that the revision rates of UKA in the long term are artificially inflated.

With improved satisfaction and comparable outcomes, UKA is a promising procedure for AMOA, with excellent functional improvement. However, the selection of the procedure should be based on the surgeon and centre’s experience, patient activity levels, and expectations. Patient selection is an important factor that can influence the outcomes after UKA or TKA. Although medial UKA combined with patellofemoral or lateral UKA is being explored as an alternative to TKA for extended indications (fixed flexion deformities, advanced patellofemoral wear, or lateral wear), long-term outcomes in these patients are not clear [44, 45]. Studies have demonstrated inferior results with combined procedures for OA of the knee [46]. Diagnostic criteria to define isolated medial OA should be adhered to strictly, to choose the right procedure.

With the number of UKAs performed increasing worldwide, further research into the outcomes and patient satisfaction comparing UKA and TKA is warranted. Large-volume or multi-centre randomized controlled trials will be required to prove the superiority of either procedure in the management of AMOA.

## Conclusion

Unicompartmental knee arthroplasty for AMOA was associated with improved patient satisfaction compared to TKA. Although PROMs were in favour of UKA over TKA with statistical significance, the clinical relevance of the differences must be interpreted carefully. Clinically comparable results with similar early complication and revision rates make UKA a compelling procedure for the management of AMOA. Multicenter and randomized studies comparing the two procedures are warranted.
